# Comparison of Drug-Coated Balloon Angioplasty vs. Drug-Eluting Stent Implantation for Drug-Eluting Stent Restenosis in the Routine Clinical Practice: A Meta-Analysis of Randomized Controlled Trials

**DOI:** 10.3389/fcvm.2021.766088

**Published:** 2021-12-01

**Authors:** Yong Zhu, Kesen Liu, Xiangyun Kong, Jing Nan, Ang Gao, Yan Liu, Hongya Han, Hong Li, Huagang Zhu, Jianwei Zhang, Yingxin Zhao

**Affiliations:** ^1^Department of Cardiology, Beijing Anzhen Hospital, Capital Medical University, Beijing, China; ^2^Department of Cardiology, Beijing Luhe Hospital, Capital Medical University, Beijing, China; ^3^Department of Cardiology, Beijing Tiantan Hospital, Capital Medical University, Beijing, China

**Keywords:** in-stent restenosis, drug-eluting stent, drug-eluting balloon, randomized controlled trial, target lesion revascularization

## Abstract

**Introduction:** In-stent restenosis (ISR) remains a challenging issue despite the great advance of drug-eluting stents (DES). In addition, the consensus was lacking regarding the optimal strategy for DES-ISR. Therefore, we aimed to evaluate angiographic and clinical outcomes of the two most effective treatments DES vs. drug-eluting balloon (DCB) for patients with DES-ISR.

**Methods:** This meta-analysis used the data from the randomized controlled trials (RCTs), which were identified by a systematic search in the databases of PubMed, Embase, and Cochrane Library. Target lesion revascularization (TLR) was regarded as the primary endpoint. In addition, the late angiographic outcomes and other clinical outcomes, namely, cardiac death, myocardial infarction (MI), target vessel revascularization, stent thrombosis, and major adverse cardiac events, were also included for analysis.

**Results:** Five RCTs with about 1,193 patients were included in this meta-analysis for the analysis. For the primary endpoint, the overall pooled outcomes suggested repeat DES implantation was associated with a significant reduction in the term of TLR compared with DCB angioplasty (risk ratio = 1.53, 95% CI 1.15–2.04, *p* = 0.003). But no significant difference in angiographic outcomes and other clinical endpoints were observed between DES and DCB. In the subgroup analysis, DCB was inferior to new-generation DES (NG-DES)/everolimus-eluting stent (EES) in the term of TLR. In addition, this non-significant trend was also noted in the subgroup of the paclitaxel-eluting stent (PES) vs. DCB. For the angiographic endpoints, EES, not PES, was associated with larger minimum lumen diameter [mean difference (MD) = −0.25, 95% CI −0.38 to −0.11, *p* = 0.0003], lower percent diameter stenosis (MD = 7.29%, 95% CI 2.86–11.71%, *p* = 0.001), and less binary restenosis (OR = 2.20, 95% CI 1.18–4.11, *p* = 0.01). But NG-DES/EES was comparable to DCB in cardiac death, MI, and stent thrombosis.

**Conclusions:** For the patients with DES-ISR, treatment with DES, especially NG-DES/EES could reduce the risk of TLR significantly compared to DCB at long-term follow-up.

## Introduction

Drug-eluting stents (DES) were widely used to treat ischemic coronary artery disease (CAD) and it has been recommended as the default choice in patients undergoing percutaneous coronary intervention (PCI) (1–3). Although the antirestenotic performance of DES improves significantly compared to bare-metal stents (BMS), 5–10% of patients treated with DES still suffer from restenosis, especially in complex clinical and anatomic settings (4, 5). Several previous studies also demonstrated patients treated for DES-in-stent restenosis (ISR) may have a worse long-term prognosis compared to these patients with BMS-ISR (6, 7). Therefore, DES restenosis after PCI has become an important and challenging issue in routine clinical practice.

With consistent improvement in the technique, various treatment strategies were performed and tested in those patients with DES-ISR, such as balloon angioplasty, cutting balloon, vascular brachytherapy, rotablation, DES implantation, drug-coated balloon (DCB) angioplasty, and excimer laser (8, 9). Among those strategies, repeat DES implantation and DCB angioplasty have been regarded as the most effective therapeutic options and recommended by current guidelines (Class I, Level A) (3). Although several randomized controlled trials (RCTs) have been designed to compare outcomes of DES vs. DCB in patients presenting with DES-ISR (10–14), few of them were powered for clinical endpoints indeed. In addition, the conclusions obtained from the RCTs were controversial and considerable heterogeneity existed in various aspects of those RCTs such as characteristic of participants, type of restenotic stent, and generation of DES used in the repeat stenting arm (10–14). Furthermore, recent strong pieces of evidence suggested the risk of death increased significantly beyond the 1st year in patients treated with paclitaxel-coated devices for peripheral artery disease (15), which raised concerns on DCB in the field of coronary intervention because almost all DCB used in the routine clinical practice were coated with paclitaxel.

Against this background, a comprehensive meta-analysis of RCTs was performed by us to investigate the angiographic and clinical outcomes of repeat DES vs. DCB angioplasty in patients presenting with DES-ISR.

## Methods

This meta-analysis was performed according to the preferred reporting items for systematic reviews and meta-analyses.

### Literature Search

A systematic literature search was performed by us in the databases of PubMed, Embase, and Cochrane Library to identify all relevant articles published from inception to June 19, 2021. The three types of search terms (and their similar terms) used in this study were listed as follows: in-stent restenosis OR coronary stent restenosis OR stent restenosis OR restenosis OR ISR; drug-eluting stent OR drug eluting stent OR drug-coated stent OR drug coated stent OR everolimus-eluting stent OR everolimus eluting stent OR everolimus-coated stent OR everolimus coated stent OR zotarolimus-eluting stent OR zotarolimus eluting stent OR zotarolimus-coated stent OR zotarolimus coated stent OR sirolimus-eluting stent OR sirolimus eluting stent OR sirolimus-coated stent OR sirolimus coated stent OR paclitaxel-eluting stent OR paclitaxel eluting stent OR paclitaxel-coated stent OR paclitaxel coated stent OR DES OR EES OR ZES OR SES OR PES; drug-coated balloon OR drug coated balloon OR drug-eluting balloon OR drug eluting balloon OR paclitaxel-coated balloon OR paclitaxel coated balloon OR paclitaxel-eluting balloon OR paclitaxel eluting balloon OR sirolimus-coated balloon OR sirolimus coated balloon OR sirolimus-eluting balloon OR sirolimus eluting balloon OR DCB OR PCB OR SCB. In addition, the reference list of eligible studies and review articles were reviewed by us to identify additional publications as well.

### Study Selection

Eligible studies were identified independently by two researchers (YZ and KSL) with the assistance of EndNote software according to the pre-specified PICOS criteria. The PICOS criteria were as follows: (1) patients: patients presenting with DES-ISR; (2) intervention: treatment with DCB; (3) comparison: repeat DES implantation; (4) outcomes: long-term (≥1 year) clinical endpoints and/or follow-up angiographic endpoints; and (5) study design: RCTs.

### Study Endpoints and Definitions

The primary endpoint was target lesion revascularization (TLR), which was defined as any revascularization procedure involving the target lesion. Other clinical outcomes, namely, cardiac death, myocardial infarction (MI), target vessel revascularization (TVR), stent thrombosis (definite or probable), and major adverse cardiac events (MACEs), which was a composite of death, MI, and TLR, were considered as secondary endpoints. Notably, a composite of cardiac death, target vessel MI, and clinically driven TLR reported in the RCT BIOLUX was regarded as MACEs for final analysis.

For the angiographic endpoints, in-segment measurements (the treated area plus its 5 mm proximal/distal edges), namely, minimum lumen diameter (MLD), percent diameter stenosis (DS%), and late lumen loss (LLL) were adopted for the analysis. In addition, binary restenosis, which was defined as >50% diameter stenosis in the segment, was also determined for the analysis.

### Data Extraction and Quality Assessment

The data extraction was performed by one author (YZ) with the standardized form recording the key items and verified by another researcher (XYK). For the included studies, the basic characteristics such as lead authors, publication years, period of recruitment, sample size, type of DCB/DES used, follow-up time, and reported outcomes were all collected. In addition to those, the clinical, lesion, procedural characteristics, and dual antiplatelet therapy (DAPT) protocol of the study population were also recorded by us.

Two independent researchers (YL and AG) were responsible for quality assessment. In addition, the quality of RCTs was assessed using revised Jadad's score, which is reliable and convenient. Notably, the discrepancies encountered in processes of study selection, data extraction, and quality assessment were resolved by discussion with the senior researcher (YXZ).

### Statistical Analysis

The heterogeneity between included studies was assessed by the Cochrane Q test (*p* < 0.1 indicates significance) and *I*^2^ statistic. When there was no significant heterogeneity across the studies (*p* > 0.1 and *I*^2^ < 50%), the principal measures risk ratio (RR) and mean difference (MD) with the corresponding 95% CI were calculated with the Mantel-Haenszel/inverse variance fixed-effects model. If significant heterogeneity was detected, the random-effects model was performed. In addition, sensitivity analysis or subgroup analysis was also performed approximately to further confirm the conclusions obtained. But the publication bias was not examined in this study because the eligible studies were limited (<10). All the statistical analyses included in this meta-analysis were performed using Review Manager version 5.3 (The Nordic Cochrane Center, Copenhagen, Denmark). In addition, *p* < 0.05 was considered as statistical significance.

## Results

### Study Selection

**Figure 1:**
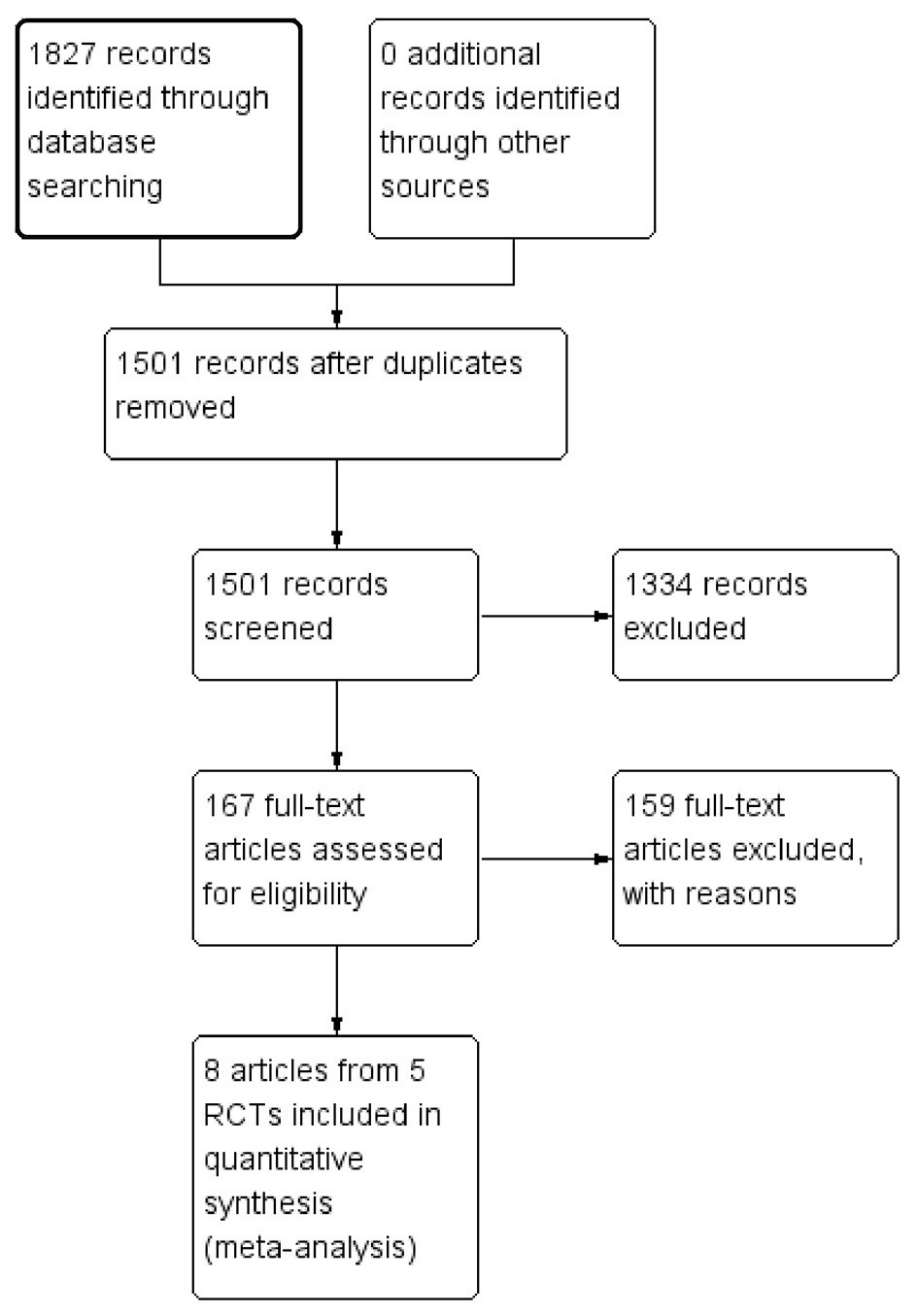
The flow chart of study selection.

As demonstrated in Figure 1, the search strategy used in this meta-analysis yielded 1,827 records, of which 1,501 records were left for screening after removing duplicates. After title and abstract screening, a total of 167 full-text articles were assessed for eligibility. In the end, eight articles from five RCTs were included for the final analysis after excluding 159 additional records by reviewing full-text articles (5, 10–14, 16, 17).

### General Characteristics of Included Studies and Patients

As present in Tables 1, 2, five multicenter RCTs (eight articles) with about 1,193 patients enrolled from 2009 to 2016 were finally included in this meta-analysis (5, 10–14, 16, 17). In addition, the included RCTs were published from 2013 to 2018. In those included studies, the angiographic follow-up period was 6–9 months, and the follow-up period for clinical outcomes ranges from 12 to 36 months. In addition to these contents, the primary endpoints/objective, inclusion criteria, and exclusion criteria of the eligible studies were also summarized and presented in Table 2.

**Table 1:** Main characteristics of the included studies.

**References**	**Data source**	**Study design**	**Multicenter**	**Region**	**Investigation time**	**Total patient (lesion)**		**DCB type**	**DES type**
		**Study quality**				**DCB**	**DES**		
Byrne et al. (10)	ISAR-DESIRE 3	RCT	Yes	Germany	2009–2011	268 (340)		PCB	PES
Kufner et al. (16)		5				137 (172)	131 (168)	(SeQuent please)	(Taxus Liberté)
Xu et al. (11)	PEPCAD China ISR Trial	RCT	Yes	China	2011–2012	215 (221)		PCB	PES
Xu et al. (17)		4				109 (113)	106 (108)	(SeQuent please)	(Taxus Liberté)
Alfonso et al. (12)	RIBS IV	RCT	Yes	Spain	2010–2013	309 (309)		PCB	EES
Alfonso et al. (5)		4				154 (154)	155 (155)	(SeQuent please)	(Xience Prime)
Jensen et al. (14)	BIOLUX	RCT	Yes	Germany	2012–2015	BMS-ISR and DES-ISR:		BTHC based PCB	BP-SES
		4		Latvia		229 (243)		(Pantera LUX)	(Orsiro)
						157 (163)	72 (80)		
Wong et al. (13)	RESTORE	RCT	Yes	South	2013–2016	172 (172)		PCB	EES
		4		Korea		86 (86)	86 (86)	(SeQuent please)	(Xience)

*RCT, randomized controlled trial; DCB, drug-coated balloon; DES, drug-eluting stents; PCB, paclitaxel-coated balloon; PES, paclitaxel-eluting stents; everolimus-eluting stents; BP-SES, biodegradable polymer sirolimus-eluting stents*.

**Table 2:** Reported outcomes and follow-up time.

**References**	**Data source**	**Follow-up time (month)**	**The primary endpoint/objective**	**Major inclusion/exclusion criteria**
Byrne et al. (10)	ISAR-DESIRE 3	Angiography: 6–8 months Clinical outcomes: 12 months	DS% in the segment	**Inclusion:** Age > 18 years; DES-ISR (DS% > 50%) with ischemic symptom or objective evidence of myocardial ischemia. **Exclusion:** Target lesion located in LM/coronary bypass graft; STEMI (within 48 h); cardiogenic shock; eGFR <30 ml/min; life expectancies <12 months; contraindication/allergy to antiplatelet therapy, paclitaxel, or stainless steel.
Kufner et al. (16)		Clinical outcomes: 36 months	The primary efficacy endpoint: TLR The primary safety endpoint: the composite of death or MI	
Xu et al. (11)	PEPCAD China ISR Trial	Angiography: 9 months Clinical outcomes: 12 months	In segment LLL	**Inclusion:** Age (18–80 years); DES-ISR (Mehran type I to III); DS% > 70 or 50% with documented myocardial ischemia. **Exclusion:** MI (within 1 week); bifurcation with SB > 2.5 mm; lesion with extensive thrombus; severe chronic HF or NYHA IV; severe VHD; stroke within 6 months; eGFR <30 ml/min.
Xu et al. (17)		Clinical outcomes: 24 months	2-year outcomes and additional subgroup analysis	
Alfonso et al. (12)	RIBS IV	Angiography:6-9 months Clinical outcomes:12 months	In-segment MLD	**Inclusion:** DES-ISR (DS% > 50%) with symptom or objective evidence of ischemia. **Exclusion:** Small vessels (<2.0 mm in diameter); long lesion (>30 mm); total occlusion; DES-ISR within 1 months; DES-ISR presenting acute MI; target lesion with obvious thrombus.
Alfonso et al. (5)		Angiography: 6-9 months Clinical outcomes: 36 months	The main objective: comparison of 3-year clinical outcome The primary endpoint: In-segment MLD	
Jensen et al. (14)	BIOLUX	Angiography: 6 months Clinical outcomes: 18 months	The primary efficacy endpoint: in-stent LLL The primary safety endpoint: TLF at 12 months	**Inclusion:** patients presenting with clinical evidence of IHD and/or a positive functional study, SAP/UAP/silent ischemia and ISR (DS% > 50%) in BMS or DES; number of ISR lesion ≤ 2; in case of 2 target lesion, both ISR lesions were treated by the same device. **Exclusion:** STEMI (within 72 h); acute cardiac decompensation or cardiogenic shock; LVEF <30%; target lesion located in LM; target vessel with thrombus; allergies to antiplatelet drugs, heparin, or similar drugs; dialysis or creatinine > 2.5 mg/dl; life expectancy <18 months; small (diameter <2 mm) or large (diameter > 4 mm) vessel; short (<6 mm) or diffuse (>28 mm) lesion.
Wong et al. (13)	RESTORE	Angiography: 9 months Clinical outcomes: 12 months	In-segment LLL	**Inclusion:** patients with DES-ISR (DS% > 50%). **Exclusion:** life expectancy **<** 12 months; contraindication to paclitaxel, everolimus, and antiplatelet drugs.

*DS%, percent diameter stenosis; TLR, target lesion revascularization; MI, myocardial infarction; DES, drug-eluting stents; ISR, in-stent restenosis; LM, left main artery; STEMI, ST-segment elevation myocardial infarction; eGFR, estimated glomerular filtration rate; LLL, late lumen loss; SB, side branch; HF, heart failure; NYHA, New York Heart Association; VHD, valvular heart disease; MLD, minimum lumen disease; TLF, target lesion failure; IHD, ischemic heart disease; SAP, stable angina pectoris; UAP, unstable angina pectoris; BMS, bare metal stents; LVEF, left ventricular ejection fraction*.

According to data obtained from the included studies (Table 3), baseline demographics, lesion, procedure characteristics, and DAPT protocol (except RIBS IV) between the DCB and DES groups were similar. In addition, from Table 3, we should realize that almost no patients with target lesions located in the left main artery were included in those eligible RCTs.

**Table 3:** Baseline demographics, lesion, and procedure characteristics.

	**ISAR-DESIRE 3**		**PEPCAD China ISR**		**RIBS IV**		**BIOLUX**		**RESTORE**	
**Variables**	**DCB**	**DES**	**DCB**	**DES**	**DCB**	**DES**	**DCB**	**DES**	**DCB**	**DES**
**Demographics**										
Age, years	67.7 ± 10.4	68.8 ± 10.0	61.8 ± 9.3	62.1 ± 9.3	66 ± 10	66 ± 10	67.2 ± 9.9	69.4 ± 8.8	67 ± 10	66 ± 9
Male, %	105 (77)	88 (67)	88 (80.7)	86 (81.1)	127 (82)	130 (84)	122 (77.7)	49 (68.1)	61 (70.9)	62 (72.1)
Diabetes mellitus, %	56 (41)	61 (47)	44 (40.4)	35 (33.0)	75 (49)	66 (43)	48 (30.6)	24 (33.3)	43 (50.0)	38 (44.2)
Hypertension, %	105 (77)	101 (77)	78 (71.6)	69 (65.1)	110 (71)	121 (78)	144 (91.7)	70 (97.2)	60 (69.8)	65 (75.6)
Hyperlipidemia, %	108 (79)	103 (79)	38 (34.9)	35 (33.0)	110 (71)	121 (78)	134 (85.4)	62 (86.1)	49 (57.0)	53 (61.6)
Current and/or smoker, %	19 (14)	15 (11)	23 (21.1)	27 (25.5)	89 (58)	87 (56)	104 (66.2)	42 (58.3)	40 (46.5)	37 (43.0)
LVEF, %	53.6 ± 9.8	54.5 ± 9.9	61.7 ± 8.5	62.3 ± 8.6	58 ± 12	59 ± 11	NA	NA	59.4 ± 8.4	59.9 ± 7.8
**Lesion**										
**Target lesion, %**										
LAD	59 (34)	50 (30)	47 (41.6)	61 (56.5)	77 (50)	71 (46)	NA	NA	48 (55.8)	52 (60.5)
LCX	54 (31)	61 (36)	21 (18.6)	13 (12.0)	27 (18)	34 (22)	NA	NA	13 (15.1)	11 (12.8)
RCA	59 (34)	56 (33)	45 (39.8)	34 (31.5)	43 (28)	45 (29)	NA	NA	24 (27.9)	21 (24.4)
LM	0	1 (1)	0	0	0	0	0	0	0	2 (2.3)
**Quantitative features**										
MLD, mm	0.97 ± 0.48	0.93 ± 0.50	0.85 ± 0.38	0.86 ± 0.41	0.79 ± 0.4	0.75 ± 0.4	1.0 ± 0.5	0.9 ± 0.5	0.63 ± 0.4	0.63 ± 0.42
DS%	64.4 ± 16.8	66.7 ± 16.5	68.26 ± 12.47	68.43 ± 13.25	69 ± 17	72 ± 15	67.2 ± 13.5	68.9 ± 14.7	77 ± 17	79 ± 13
Lesion length, mm	NA	NA	12.52 ± 6.55	13.08 ± 7.13	10.4 ± 5.6	10.7 ± 5.4	5.8 ± 4.0	7.2 ± 6.1	18.1 ± 9.7	17.4 ± 11.4
**Procedure**										
Predilation, %	139 (81)	145 (86)	112 (99.1)	107 (99.1)	NA	NA	160 (98.2)	77 (96.3)	65 (75.6)	72 (83.7)
Cutting/scoring balloon, %	2 (1)	2 (1)	NA	NA	7 (4)	5 (3)	NA	NA	NA	NA
Device length, mm	NA	NA	19.73 ± 5.88	20.12 ± 7.07	19 ± 6	19 ± 8	20.4 ± 5.0	20.5 ± 6.5	28.5 ± 14.7	25.5 ± 11.5
Device diameter, mm	NA	NA	3.06 ± 0.39	2.98 ± 0.39	NA	NA	3.2 ± 0.4	3.0 ± 0.5	2.98 ± 0.40	3.14 ± 0.35
DAPT protocol, months	>6	>6	>12	>12	>3	>12	NA	NA	>6	>6

*The baseline data of patients presenting DES-ISR was not available from BIOLUX, therefore we used the baseline data of general participant (BMS-ISR and DES-ISR) instead*.

*ISR, in-stent restenosis; DCB, drug-coated balloon; DES, drug-eluting stent; LVEF, left ventricular ejection fraction; LAD, left anterior descending artery; LCX, left circumflex artery; RCA, right coronary artery; LM, left main; MLD, minimum lumen diameter; DS%, percent diameter stenosis; DAPT, dual antiplatelet therapy; NA, not applicable; BMS, bare-mental stent*.

### DCB vs. DES for Angiographic Endpoints

**Figure 2:**
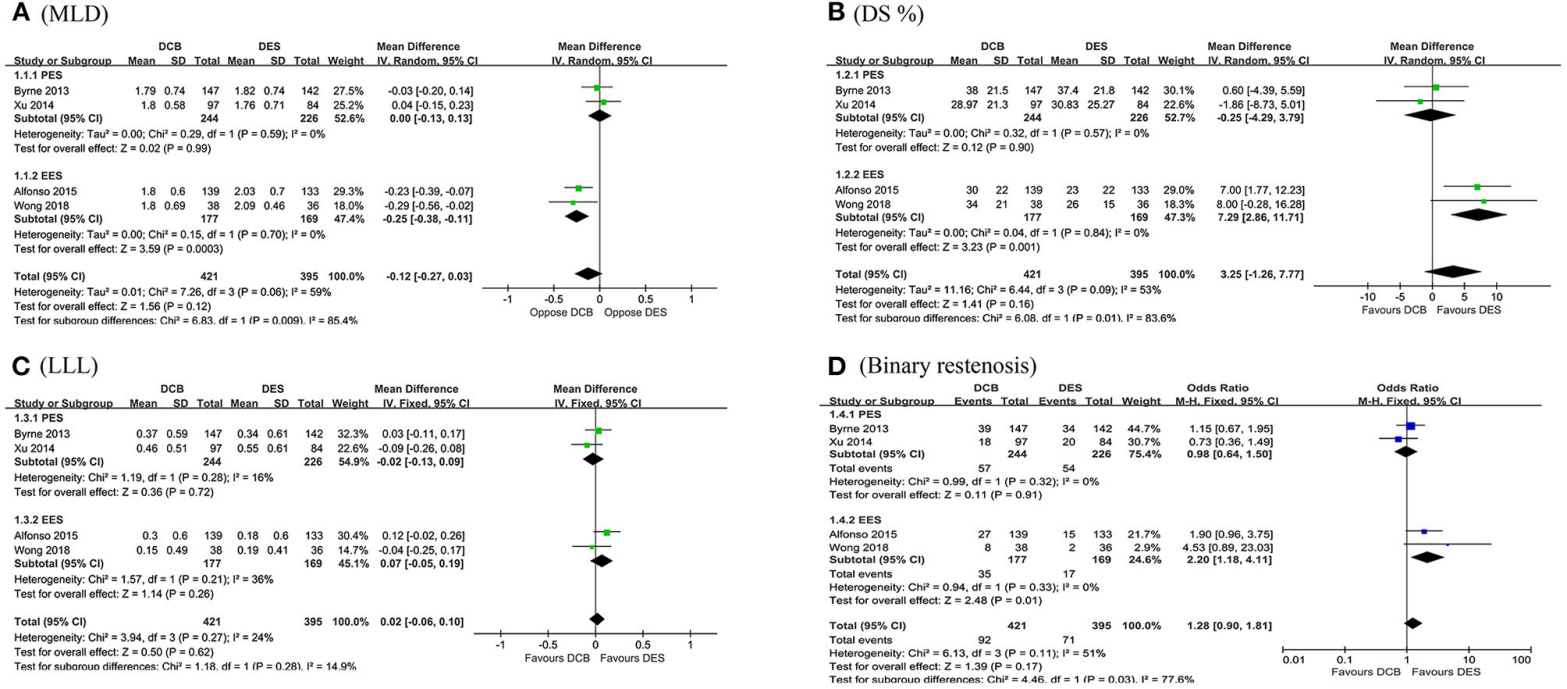
Forest plot of comparing angiographic outcomes in MLD **(A)**, DS% **(B)**, LLL **(C)**, and binary restenosis **(D)** between repeat DES implantation and DCB angioplasty. MLD, minimum lumen diameter; DS%, percent diameter stenosis; LLL, late lumen loss; DES, drug-eluting stents; DCB, drug-coated balloon; PES, paclitaxel-eluting stents; EES, everolimus-eluting stents.

In this meta-analysis, a total of 816 target lesions treated successfully by either DCB or DES were followed by angiography range from 6 to 9 months. Overall, no significant differences in MLD, DS%, LLL, and binary restenosis were detected between the DES and DCB groups. However, subsequent subgroup analysis demonstrated a significant trend toward an increment in MLD (MD = −0.25 mm, 95 CI −0.38 to −0.11 mm, *p* = 0.0003; *I*^2^ = 0%, *p* = 0.70, Figure 2A) was observed in the everolimus-eluting stents (EES) group. In addition, patients receiving EES were associated with significantly lower DS% (MD = 7.29, 95% CI 2.86–11.71, *p* = 0.001; *I*^2^ = 0%, *p* = 0.84, Figure 2B) and the risk of binary restenosis (OR = 2.20, 95% CI 1.18–4.11, *p* = 0.01; *I*^2^ = 0%, *p* = 0.33, Figure 2D) compared to the patients treated with DCB. However, no significant difference in LLL was noted between DES and DCB (Figure 2C).

### DCB vs. DES for the Primary Endpoint TLR

**Figure 3:**
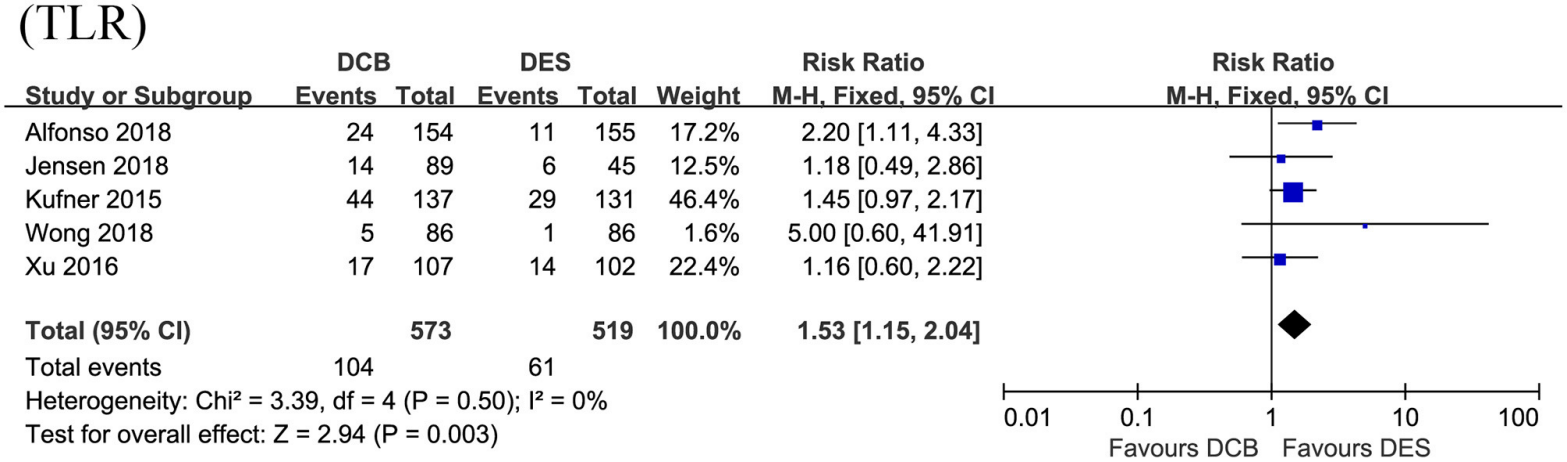
Forest plot of comparing the risk of TLR between repeat DES implantation and DCB angioplasty. TLR, target lesion revascularization; DES, drug-eluting stents; DCB, drug-coated balloon.

**Figure 4:**
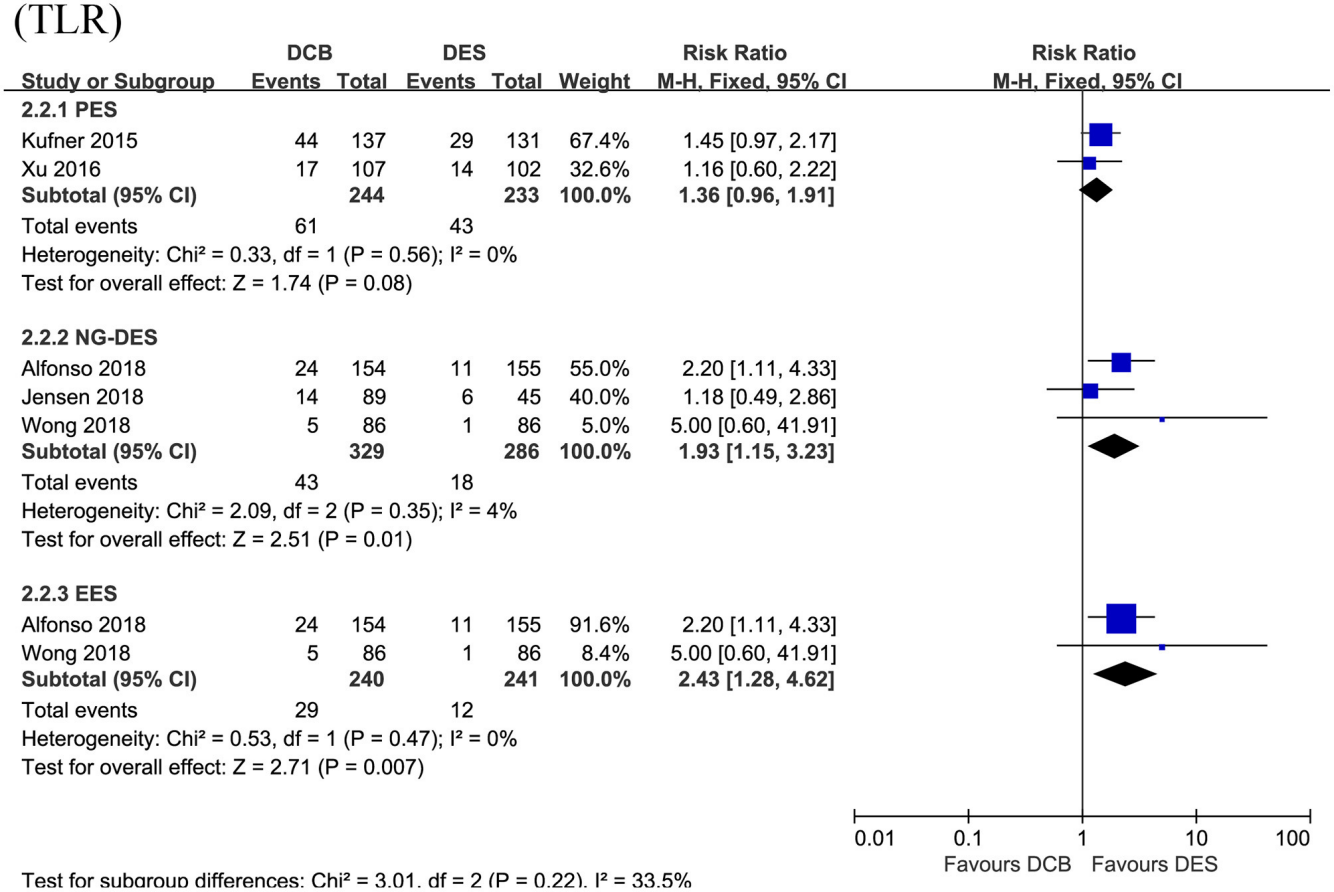
Subgroup analysis for the primary endpoint TLR according to the type of DES used. TLR, target lesion revascularization; DES, drug-eluting stents; PES, paclitaxel-eluting stents; NG-DES, new-generation DES; EES, everolimus-eluting stents.

In this section, the longest available clinical follow-up periods were considered for the analysis and the primary endpoint TLR was reported in the five RCTs including 1,092 patients. After pooling the data of 573 patients receiving DCB vs. 519 patients receiving DES, this meta-analysis revealed repeat DES implantation was associated with reduced TLR compared to DCB angioplasty in patients presenting with DES-ISR (RR = 1.53, 95% CI 1.15–2.04, *p* = 0.003, Figure 3), with low heterogeneity across the trials (*I*^2^ = 0%, *p* = 0.50). In the subgroup analysis, a strong trend toward a decrease in TLR (RR = 1.36, 95% CI 0.96–1.91, *p* = 0.08; *I*^2^ = 0%, *p* = 0.56, Figure 4) was even noted in paclitaxel-eluting stent (PES) group, although this difference was not statistically significant compared to DCB.

### DCB vs. DES for Secondary Outcomes

**Figure 5:**
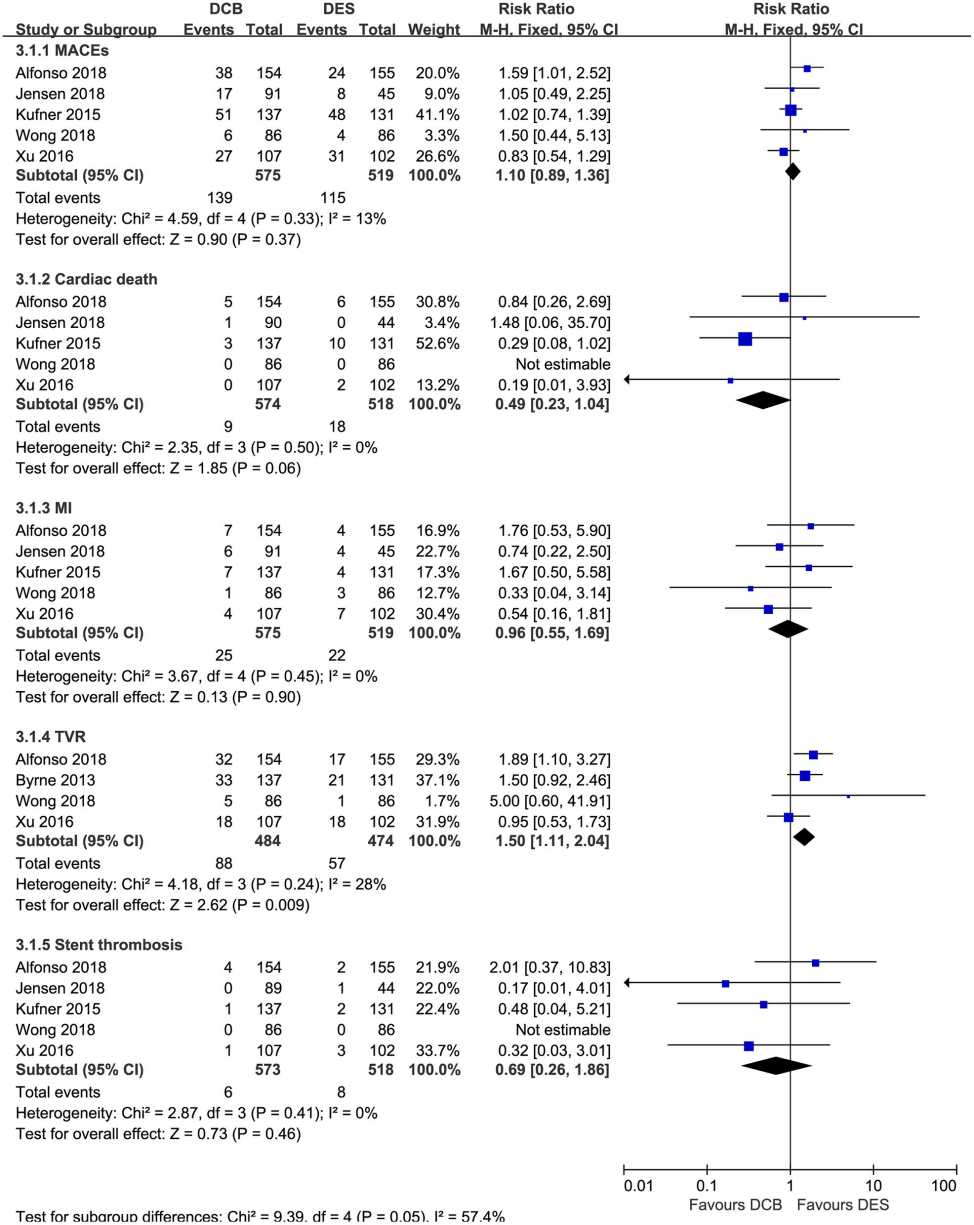
Forest plot comparing secondary outcomes between repeat DES implantation and DCB angioplasty. DES, drug-eluting stents; DCB, drug-coated balloon; MACEs, major adverse cardiac events; MI, myocardial infarction; TVR, target vessel revascularization.

For the secondary outcomes, as demonstrated in Figure 5, no significant differences in MACEs, cardiac death, MI, and stent thrombosis were noted between the DCB group and the DES group in this meta-analysis. However, repeat DES implantation may be superior to DCB angioplasty in reducing the risk of TVR (RR = 1.50, 95% CI 1.11–2.04, *p* = 0.009; *I*^2^ = 28%, *p* = 0.24, Figure 5). In the subgroup analysis (Table 4), we also found repeat revascularization with EES, compared with DCB angioplasty, could reduce the risk of MACEs (RR = 1.58, 95% CI 1.03–2.43, *p* = 0.04). However, in terms of cardiac death, DES especially PES may be inferior to DCB.

**Table 4:** Subgroup analysis for secondary outcomes.

	**Number of studies**	**RR (95% CI)**	***P* for test**	***I* ^**2**^**	***P* for heterogeneity**
**MACEs**					
PES	2	0.94 (0.73, 1.22)	0.65	0%	0.46
NG-DES	3	1.43 (0.99, 2.08)	0.06	0%	0.65
Only EES	2	1.58 (1.03, 2.43)	**0.04**	0%	0.93
**Cardiac death**					
PES	2	0.27 (0.08, 0.86)	**0.03**	0%	0.81
NG-DES	3	0.90 (0.30, 2.69)	0.86	0%	0.74
Only DES	2	0.84 (0.26, 2.69)	0.77	-	-
**MI**					
PES	2	0.95 (0.42, 2.16)	0.91	40%	0.20
NG-DES	3	0.97 (0.45, 2.09)	0.94	0%	0.37
Only EES	2	1.15 (0.42, 3.12)	0.79	39%	0.20
**TVR**					
PES	2	1.25 (0.86, 1.82)	0.25	25%	0.25
NG-DES (EES)	2	2.07 (1.22, 3.50)	**0.007**	0%	0.41
**Stent thrombosis**					
PES	2	0.38 (0.07, 1.95)	0.25	0%	0.81
NG-DES	3	1.09 (0.30, 4.00)	0.90	46%	0.17
Only EES	2	2.01 (0.37, 10.83)	0.42	-	-

*RR, risk ratio; CI, confidence interval; MACEs, major adverse cardiac events; PES, paclitaxel-eluting stents; NG-DES, new-generation drug-eluting stent; EES, everolimus-eluting stents; MI, myocardial infarction; TVR, target vessel revascularization*.

### Publication Bias and Sensitivity Analysis

In this meta-analysis, the publication bias was not assessed by funnel plot because of the limited number of included studies. The sensitivity analysis, which was performed by sequentially omitting one trial at a time, also confirmed that DES outperformed DEB in terms of the primary endpoints TLR. However, after excluding the study from Alfonso et al. the difference in TVR loses significance (RR = 1.34, 95% CI 0.93–1.94, *p* = 0.12).

## Discussion

This meta-analysis comprehensively compared the angiographic and clinical endpoints of the two different strategies (DCB vs. DES) for patients presenting with DES-ISR in routine clinical practice. In addition, the major findings were listed as follows: (1) EES, not PES, was superior to DCB in angiographic endpoints, with larger MLD, lower DS%, and less binary restenosis. (2) For the clinical endpoints, the overall pooled outcomes demonstrated revascularization with DES significantly reduced the risk of TLR and TVR compared to DCB angioplasty. (3) In addition to TLR and TVR, EES was also associated with lower MACEs than DCB in the subgroup analysis.

Despite the great advance in primary and secondary prevention, CAD remains the leading cause of mortality worldwide (18, 19). PCI based on DES is the most commonly used strategy for myocardial revascularization in patients with CAD at present (1, 3). Although the efficacy of DES in the prevention of restenosis improves significantly compared to BMS, DES-ISR still develops in 5–10% of patients after DES deployment (5, 20). In addition, the pathophysiology underlying DES-ISR may be more complex. In addition to neointimal hyperplasia and stent under-expansion, which were considered as the dominant risk factors for BMS-ISR, neo-atherosclerosis seems to be another factor contributing to DES-ISR (8, 9). Furthermore, DES-ISR continues to be a therapeutic challenge and trials designed to determine the optimal treatment strategy for patients with DES-ISR were limited.

Although repeat stenting with DES and DCB angioplasty has been recommended by current guidelines, the consensus was lacking regarding the best treatment for DES-ISR. Bajraktari et al. performed a meta-analysis of seven studies (three of which were RCTs) and reported DEB was comparable to DES in terms of clinical outcomes (MACEs, cardiac death, MI, stent thrombosis, TLR, and TVR) for the treatment of DES-ISR (21). However, Giacoppo et al. reported, in the meta-analysis of individual patient data from all available RCTs (DAEDALUS), DCB angioplasty is significantly associated with a higher risk of TLR compared to repeat stenting with DES for the treatment of DES-ISR at 3-year follow-up (22). In addition, the primary safety endpoint (a composite of all-cause mortality, MI, and target lesion thrombosis) was similar between the two groups (22). In consistent with the study DAEDALUS, this study also confirmed repeat DES implantation was superior to DCB angioplasty in terms of TLR. Moreover, this trend still persisted when we compared PES with DCB, but it was marginally significant. To extend previous studies, this meta-analysis further confirmed EES, not PES, outperforms DCB in terms of MLD, DS%, and binary restenosis.

Our conclusions were reliable and stable because we only included RCTs and the sensitivity analysis and subgroup analysis were performed. But we should keep in mind our conclusions may not generalize to the patients with target lesions located in the left main artery or aorto-ostial coronary. Because few such patients were included in this study. Therefore, multicenter, prospective, and RCTs are needed to figure out this knowledge gap.

### Future Perspectives

DES, especially new-generation (NG-DES)/EES, was superior to DCB in reducing the risk of TLR in this meta-analysis. But treatment with DCB for DES-ISR could avoid multilayers of metal stents in the coronary artery, which poses difficulty for further treatment of recurrent restenosis and is associated with a poor prognosis (23–25). In addition, DCB may be more suitable for patients who are intolerable of long-term DAPT or at a high risk of bleeding (26, 27). Therefore, further investigating and refining DCB technology were warranted. First, future RCTs should focus on the strategy of DCB angioplasty combined with neointimal modification by scoring/cutting balloon, rotablation, and excimer laser. In addition, Kufner et al. have confirmed in patients with DES-ISR, neointimal modification with scoring balloon before DCB was superior over DCB angioplasty alone (28). Second, to extend the previous study from Ali et al. (29), RCTs with large sample sizes and extended clinical follow-up time are required to verify the efficacy and safety of sirolimus-coated balloon (SCB) in patients with DES-ISR. Third, compared to DCB, bioresorbable vascular scaffolds (BVS) could prevent early recoil without adding another layer of metal. Alfonso et al. have confirmed BVS was similar to DCB but inferior to EES in late angiographic and clinical results for the treatment of any ISR in RIBS trial VI (30). In addition, Moscarella et al. also reported BVS was associated with a numerically higher rate of device-oriented cardiovascular events (DOCE) compared with EES while a similar rate compared to DCB for the treatment for any ISR in the BIORESOLVE-ISR Study (31). But the value of BVS in the scenario of DES-ISR remains unsettled and future trials are needed to investigate it.

### Limitations

Several limitations should be acknowledged in this meta-analysis. First, the type of restenotic DES, generation of DES used, time from implantation to admit for DES-ISR, and antithrombotic strategy after intervention for DES-ISR were not uniformly across the included RCTs, which may cause heterogeneity. Second, this meta-analysis only included five RCTs. Therefore, some subgroups were not powered to detect differences and publication bias was not evaluated. Third, this meta-analysis was based on the study level rather than the patient level. In addition, the ratio of follow-up angiography at the scheduled time was suboptimal and the exact reasons were not reported. Finally, the follow-up time for the main analysis was limited up to 3 years in this meta-analysis. Future studies with an extended follow-up duration could provide an additional value to assess the efficacy and safety of DES vs. DCB for DES-ISR.

## Conclusion

Angioplasty with DCB was moderately less effective than repeat DES implantation in reducing the TLR for patients with coronary DES-ISR at long-term follow-up. In addition, this trend was more obvious when compared with NG-DES/EES. In addition, repeat stenting with EES could also provide better angiographic outcomes than DCB angioplasty. To confirm our findings, RCTs with a large sample size and extended follow-up duration are required in the future.

## Data Availability Statement

The original contributions presented in the study are included in the article/supplementary material, further inquiries can be directed to the corresponding authors.

## Author Contributions

Data analysis, interpretation, and manuscript writing were performed by YZhu. Literature search, study selection, data extraction, and quality assessment were performed by YZhu, KL, XK, YL, AG, and YZha. YZha, JZ, and HH were responsible for the conception and design of the study. JN and HL revised the manuscript carefully. All authors read and approved the final manuscript.

## Funding

This work was supported by the grant from the National Key Research and Development Program of China (2017YFC0908800), the Beijing Municipal Administration of Hospitals Ascent Plan (DFL20150601) and Mission Plan (SML20180601), and the Beijing Municipal Health Commission Project of Science and Technology Innovation Center (PXM2019_026272_000006) (PXM2019_026272_000005).

## Conflict of Interest

The authors declare that the research was conducted in the absence of any commercial or financial relationships that could be construed as a potential conflict of interest.

## Publisher's Note

All claims expressed in this article are solely those of the authors and do not necessarily represent those of their affiliated organizations, or those of the publisher, the editors and the reviewers. Any product that may be evaluated in this article, or claim that may be made by its manufacturer, is not guaranteed or endorsed by the publisher.

## Abbreviations

DES: drug-eluting stents
CAD: coronary artery disease
PCI: percutaneous coronary intervention
BMS: bare-metal stents
DCB: drug-coated balloon
RCTs: randomized controlled trials
TLR: target lesion revascularization
MI: myocardial infarction
TVR: target vessel revascularization
MACEs: major adverse cardiac events
MLD: minimum lumen diameter
DS%: percent diameter stenosis
LLL: late lumen loss
RR: risk ratio
MD: mean difference
CI: confidence interval
EES: everolimus-eluting stents
PES: paclitaxel-eluting stents
NG-DES: new-generation DES
SCB: sirolimus-coated balloon
BVS: bioresorbable vascular scaffolds.

## References

[1] Stefanini GG Holmes DR Jr. Drug-eluting coronary-artery stents. N Engl J Med. (2013) 368:254–65. 10.1056/NEJMra1210816 23323902

[2] Serrador Frutos AM Jiménez-Quevedo P Pérez De Prado A PanÁlvarez-Ossorio M. Spanish cardiac catheterization and coronary intervention registry. 26th Official report of the spanish society of cardiology working group on cardiac catheterization and interventional cardiology (1990-2016). Rev Esp Cardiol (Engl Ed). (2017) 70:1110–20. 10.1016/j.rec.2017.10.017 29113720

[3] Neumann FJ Sousa-Uva M Ahlsson A Alfonso F Banning AP Benedetto U . 2018 ESC/EACTS Guidelines on myocardial revascularization. Eur Heart J. (2019) 40:87–165. 10.1093/eurheartj/ehy855 30165437

[4] Basavarajaiah S Naganuma T Latib A Sticchi A Ciconte G Panoulas V . Treatment of drug-eluting stent restenosis: comparison between drug-eluting balloon versus second-generation drug-eluting stents from a retrospective observational study. Catheter Cardio Interv. (2016) 88:522–8. 10.1002/ccd.26368 26715370

[5] Alfonso F Pérez-Vizcayno MJ Cuesta J García Del Blanco B García-Touchard A López-Mínguez JR . 3-Year clinical follow-up of the RIBS IV clinical trial. JACC Cardiovasc Interv. (2018) 11:981–91. 10.1016/j.jcin.2018.02.037 29798776

[6] Steinberg DH Gaglia MA Pinto Slottow TL Roy P Bonello L De Labriolle A . Outcome differences with the use of drug-eluting stents for the treatment of in-stent restenosis of bare-metal stents versus drug-eluting stents. Am J Cardiol. (2009) 103:491–5. 10.1016/j.amjcard.2008.09.107 19195508

[7] Alfonso F Pérez-Vizcayno MJ García Del Blanco B García-Touchard A López-Mínguez J Masotti M . Everolimus-eluting stents in patients with bare-metal and drug-eluting in-stent restenosis. Circulation Cardiovasc Interv. (2016) 9:e003479. 10.1161/CIRCINTERVENTIONS.115.003479 27412868

[8] Alfonso F Byrne RA Rivero F Kastrati A. Current treatment of in-stent restenosis. J Am Coll Cardiol. (2014) 63:2659–73. 10.1016/j.jacc.2014.02.545 24632282

[9] Shlofmitz E Iantorno M Waksman R. Restenosis of drug-eluting stents. Circulation Cardiovasc Interv. (2019) 12:e007023. 10.1161/CIRCINTERVENTIONS.118.007023 31345066

[10] Byrne RA Neumann F Mehilli J Pinieck S Wolff B Tiroch K . Paclitaxel-eluting balloons, paclitaxel-eluting stents, and balloon angioplasty in patients with restenosis after implantation of a drug-eluting stent (ISAR-DESIRE 3): a randomised, open-label trial. Lancet. (2013) 381:461–7. 10.1016/S0140-6736(12)61964-3 23206837

[11] Xu B Gao RL Wang J'An Yuejin Y Chen SL Liu B . A prospective, multicenter, randomized trial of paclitaxel-coated balloon versus paclitaxel-eluting stent for the treatment of drug-eluting stent in-stent restenosis: results from the PEPCAD China ISR trial. JACC Cardiovasc Interv. (2014) 7:204–11. 10.1016/j.jcin.2013.08.011 24556098

[12] Alfonso F Perez-Vizcayno MJ Cardenas A Garcia DBB Garcia-Touchard A Lopez-Minguez JR . A Prospective randomized trial of drug-eluting balloons versus everolimus-eluting stents in patients with in-stent restenosis of drug-eluting stents: the RIBS IV randomized clinical trial. J Am Coll Cardiol. (2015) 66:23–33. 10.1016/j.jacc.2015.04.063 26139054

[13] Wong YTA Kang DY Lee JB Rha SW Hong YJ Shin ES . Comparison of drug-eluting stents and drug-coated balloon for the treatment of drug-eluting coronary stent restenosis: a randomized RESTORE trial. Am Heart J. (2018) 197:35–42. 10.1016/j.ahj.2017.11.008 29447782

[14] Jensen CJ Richardt G Tölg R Erglis A Skurk C Jung W . Angiographic and clinical performance of a paclitaxel-coated balloon compared to a second-generation sirolimus-eluting stent in patients with in-stent restenosis: the BIOLUX randomised controlled trial. EuroIntervention. (2018) 14:1096–103. 10.4244/EIJ-D-17-01079 29808819

[15] Katsanos K Spiliopoulos S Kitrou P Krokidis M Karnabatidis D. Risk of death following application of paclitaxel-coated balloons and stents in the femoropopliteal artery of the leg: a systematic review and meta-analysis of randomized controlled trials. J Am Heart Assoc. (2018) 7:e011245. 10.1161/JAHA.118.011245 30561254 PMC6405619

[16] Kufner S Cassese S Valeskini M Neumann FJ Schulz-Schupke S Hoppmann P . Long-term efficacy and safety of paclitaxel-eluting balloon for the treatment of drug-eluting stent restenosis: 3-year results of a randomized controlled trial. JACC Cardiovasc Interv. (2015) 8:877–84. 10.1016/j.jcin.2015.01.031 26003022

[17] Xu B Qian J Ge J Wang J Chen F Chen J . Two-year results and subgroup analyses of the PEPCAD China in-stent restenosis trial: a prospective, multicenter, randomized trial for the treatment of drug-eluting stent in-stent restenosis. Catheter Cardio Interv. (2016) 87:624–9. 10.1002/ccd.26401 26775079

[18] Da Silva A Caldas APS Hermsdorff HHM Bersch-Ferreira ÂC Torreglosa CR Weber B . Triglyceride-glucose index is associated with symptomatic coronary artery disease in patients in secondary care. Cardiovasc Diabetol. (2019) 18:89. 10.1186/s12933-019-0893-2 31296225 PMC6625050

[19] Sun Z Cao Y Li H. Multislice computed tomography angiography in the diagnosis of coronary artery disease. J Geriatr Cardiol. (2011) 8:104–13. 10.3724/SP.J.1263.2011.00104 22783294 PMC3390077

[20] Bønaa KH Mannsverk J Wiseth R Aaberge L Myreng Y Nygård O . Drug-eluting or bare-metal stents for coronary artery disease. N Engl J Med. (2016) 375:1242–52. 10.1056/NEJMoa1607991 27572953

[21] Bajraktari G Jashari H Ibrahimi P Alfonso F Jashari F Ndrepepa G . Comparison of drug-eluting balloon versus drug-eluting stent treatment of drug-eluting stent in-stent restenosis: a meta-analysis of available evidence. Int J Cardiol. (2016) 218:126–35. 10.1016/j.ijcard.2016.05.040 27232924

[22] Giacoppo D Alfonso F Xu B Claessen BEPM Adriaenssens T Jensen C . Paclitaxel-coated balloon angioplasty vs. drug-eluting stenting for the treatment of coronary in-stent restenosis: a comprehensive, collaborative, individual patient data meta-analysis of 10 randomized clinical trials (DAEDALUS study). Eur Heart J. (2020) 41:3715–28. 10.1093/eurheartj/ehz861 31511862 PMC7706792

[23] Singh AD Singal AK Mian A Kapadia SR Hedrick DP. Kanaa'N A . Recurrent drug-eluting stent in-stent restenosis: a state-of-the-art review of pathophysiology, diagnosis, and management. Cardiovasc Revasc Med. (2020) 21:1157–63. 10.1016/j.carrev.2020.01.005 31959561

[24] Wang G Zhao Q Chen Q Zhang X Tian L Zhang X. Comparison of drug-eluting balloon with repeat drug-eluting stent for recurrent drug-eluting stent in-stent restenosis. Coron Artery Dis. (2019) 30:473–80. 10.1097/MCA.0000000000000784 31464729 PMC6791562

[25] Kawamoto HM Ruparelia NMD Latib AM Miyazaki TM Sato KM Mangieri AM . Drug-coated balloons versus second-generation drug-eluting stents for the management of recurrent multimetal-layered in-stent restenosis. JACC Cardiovasc interv. (2015) 8:1586–94. 10.1016/j.jcin.2015.04.032 26386758

[26] Roncalli J Godin M Boughalem K Shayne J Piot C Huret B . Paclitaxel drug-coated balloon after bare-metal stent implantation, an alternative treatment to drug-eluting stent in high bleeding risk patients (the panelux trial). J Invasive Cardiol. (2019) 31:94–100. 30927531 10.25270/jic/18.00272

[27] Scheller B. Antithrombozytäre therapie und PCI. Herz. (2014) 39:819–21. 10.1007/s00059-014-4158-2 25347951

[28] Kufner S Joner M Schneider S Tolg R Zrenner B Repp J . Neointimal modification with scoring balloon and efficacy of drug-coated balloon therapy in patients with restenosis in drug-eluting coronary stents: a randomized controlled trial. JACC Cardiovasc Interv. (2017) 10:1332–40. 10.1016/j.jcin.2017.04.024 28683939

[29] Ali RM Abdul Kader MASK Wan Ahmad WA Ong TK Liew HB Omar A . Treatment of coronary drug-eluting stent restenosis by a sirolimus- or paclitaxel-coated balloon. JACC Cardiovasc Interv. (2019) 12:558–66. 10.1016/j.jcin.2018.11.040 30898253

[30] Alfonso F Cuesta J Pérez-Vizcayno MJ García Del Blanco B Rumoroso JR Bosa F . Bioresorbable vascular scaffolds for patients with in-stent restenosis: the RIBS VI study. JACC Cardiovasc interv. (2017) 10:1841–51. 10.1016/j.jcin.2017.06.064 28866036

[31] Moscarella E Tanaka A Ielasi A Cortese B Coscarelli S De Angelis MC . Bioresorbable vascular scaffold versus everolimus-eluting stents or drug eluting balloon for the treatment of coronary in-stent restenosis: 1-Year follow-up of a propensity score matching comparison (the BIORESOLVE-ISR Study). Catheter Cardio Inter. (2018) 92:668–77. 10.1002/ccd.27473 29356269

